# Analysis of the Incidence and Risk Factors of Precocious Puberty in Girls during the COVID-19 Pandemic

**DOI:** 10.1155/2022/9229153

**Published:** 2022-09-28

**Authors:** Dongxia Fu, Tao Li, Yingxian Zhang, Huizhen Wang, Xue Wu, Yongxing Chen, Bingyan Cao, Haiyan Wei

**Affiliations:** ^1^Department of Endocrinology and Inborn Error of Metabolism, Children's Hospital Affiliated to Zhengzhou University, Henan Children's Hospital, Zhengzhou Children's Hospital, Zhengzhou, Henan 450000, China; ^2^Department of Endocrinology and Inborn Error of Metabolism, Beijing Children's Hospital Affiliated to Capital Medical University, Beijing 100000, China

## Abstract

Home quarantine due to the global coronavirus disease 2019 (COVID-19) pandemic has had a significant impact on children. Lifestyle changes have led to an increase in precocious puberty (PP) among girls, and the underlying risk factors for this remain unclear. Thus, we aimed to assess the influence of environmental, genetic, nutritional, and other lifestyle factors on the risk of PP in girls. We evaluated the incidence of new-onset PP in girls during home quarantine for COVID-19 and analyzed the potential risk factors. This was a retrospective questionnaire and medical record-based study involving 22 representative medical units from 13 cities in Henan Province, China. Girls with new-onset PP (central precocious puberty, 58; premature thelarche, 58; age, 5–9 years) between February 2020 and May 2020 were included, along with 124 healthy, age-matched controls. The number of new-onset PP cases reported during the study period was compared with that reported between February and May in 2018 and 2019. Patients' families completed a questionnaire to assess potential risk factors. There was a 5.01- and 3.14-fold increase in the number of new-onset PP cases from 2018 to 2020 and from 2019 to 2020, respectively; the differences were statistically significant (*p* < 0.01). High-risk factors for PP included longer time spent using electronic devices, decreased exercise time, higher body mass index, vitamin D deficiency, young age (<12 years) of mother during menarche, consumption of fried food and processed meat, residence in rural areas, and consumption of off-season fruits. Thus, we found that lifestyle changes caused due to the COVID-19 pandemic led to a significant increase in PP in girls. Management of the risk factors identified in this study may help in PP prevention.

## 1. Introduction

Precocious puberty (PP) is defined as the onset of secondary sexual characteristics at an unusually early age. It mainly occurs in girls and manifests as breast development before 8 years of age or menarche before 10 years of age [1, 2]. Depending on the involvement of the activated hypothalamic-pituitary-gonadal (HPG) axis, PP has two forms: (1) gonadotropin-dependent PP (GDPP) or central PP (CPP) and (2) gonadotropin-independent precocious puberty or peripheral PP (PPP). The most frequently encountered form of CPP (>90% in girls) is idiopathic CPP [2], and it is diagnosed based on exclusion. There are several PPP variants based on clinical traits; the most common one is premature thelarche (PT). Some cases of PT convert to CPP for unknown reasons [3]. CPP significantly affects the physical and mental health of adolescents and is associated with long-term hazards. CPP can cause premature fusion of the epiphyses, resulting in short stature in adulthood, psychological and social problems due to changes in body shape or early menarche, and psychological problems in parents/guardians. Studies have also shown that prepubertal menarche is associated with reproductive tumors and breast cancer in adulthood [4, 5].

The incidence of PP is increasing worldwide [6–9]. Home quarantine due to the global coronavirus disease 2019 (COVID-19) pandemic has had a significant impact on people's daily lives, including changes in dietary habits, exercise patterns, work and rest routines, and access to medical treatment. This induced the China government to adopt severely restrictive measures in order to reduce the risk of contagion, forcing most of the children to stop going to school, restrain from leisure activities, attend online classes at home, and remain at home for several weeks. Indeed, quarantine was the most effective method to reduce the infection risk. The number of patients presenting with PP increased dramatically after lockdown restrictions were eased. The Children's Hospital of Zhejiang University reported that the number of outpatients with new-onset PP, simple breast development, and fast-paced puberty from January to March 2020 was 4.6 times, 5.24 times, and 3.51 times that of the same period in the previous year, respectively [10]. A study from Italy reported similar findings [11]. During the COVID-19 pandemic, the number of children with new-onset CPP increased and the pubertal progress of previously diagnosed patients with CPP accelerated. However, the studies mentioned above were all single-center studies; therefore, there may have been patient-selection bias. It is necessary to conduct a multicenter survey to better understand the incidence of PP among children and girls under long-term home quarantine during the pandemic.

Previous studies have shown that PP may be related to the environment [12], nutrition [3], and genetics [13]. During the pandemic, lifestyle changes, decreased performance of outdoor activities, weight gain, significant increase in the number of online classes, and changes in melatonin (MT), serotonin, leptin, and kisspeptin levels may have affected the incidence of PP; this needs to be studied further.

This study investigated the number of girls with new-onset PP who visited 22 hospitals in Henan Province between February and May 2020, after the COVID-19 pandemic restrictions were eased in China. The prevalence of new-onset PP was compared with that in the same period in 2018 and 2019. We aimed to assess the influence of environmental, genetic, nutritional, and other lifestyle factors on the risk of PP in girls.

## 2. Materials and Methods

### 2.1. Inclusion/Exclusion Criteria

The inclusion criteria were as follows: (1) female sex, (2) diagnosis of CPP and PT according to the respective diagnostic criteria, (3) residence in Henan Province for >3 years, (4) informed consent obtained from the guardians and children, and (5) willingness to cooperate with questionnaire surveys and related examinations. The exclusion criteria were as follows: (1) CPP caused by intracranial lesions; (2) unwillingness to cooperate with questionnaire surveys or clinical auxiliary examinations; (3) previous diagnosis of PP, regardless of treatment; and (4) PPP with a clear cause, such as exogenous drug abuse.

### 2.2. Multicenter Survey

Trends in PP prevalence in female patients from 22 centers in Henan Province during the COVID-19 pandemic were analyzed. Our questionnaire was distributed in 22 representative medical units from 13 cities in various regions of Henan Province, including Henan Children's Hospital, the First Affiliated Hospital of Xinxiang Medical College, Kaifeng Children's Hospital, Qinyang People's Hospital, Hebi First People's Hospital, Henan Provincial Hospital of Traditional Chinese Medicine, Shangqiu People's Hospital, Puyang Maternity and Child Care Hospital, Anyang Maternal and Child Health Care Hospital, Xinxiang Central Hospital, Anyang People's Hospital, Pingdingshan Second People's Hospital, Xuchang Central Hospital, PLA 371 Hospital, Zhoukou People's Hospital, Luoyang Maternal and Child Health Hospital, PLA 152 Central Hospital, Huaxian People's Hospital, Xiayi People's Hospital, The First Affiliated Hospital of Henan University of Science and Technology, Luohe Children's Hospital, and Nanyang Central Hospital. The ICD-10 disease code and “precocious puberty” were used as the search terms to screen children diagnosed with new-onset PP from February to May in the 3 study years (2018–2020). All the electronic medical records from these units were carefully checked, the number of outpatient visits in each unit during the same period was recorded, and new female patients were screened for PP. With retrospective study, we analyzed the number of girls with new-onset PP in different periods and calculated the prevalence rate of new-onset PP (the number of girls with new-onset PP/the total number of outpatient visits in the same period) and change rate (CR = ratio of the prevalence of female cases of new-onset PP in different time periods). A CR of <0.5 and >3 indicated inhibited and stimulated diseases [10]. All eligible patients in the multicenter survey were included with a waiver of consent.

### 2.3. Participants

Girls with new-onset PP (aged 5–9 years) who visited Henan Children's Hospital/Children's Hospital affiliated to Zhengzhou University between February and May 2020 were recruited to investigate the factors that influence new-onset PP in girls. Patients with PP were classified into the CPP and PT groups according to bone age (BA) status, uterine/ovarian color Doppler ultrasound examination, gonadotropin-releasing hormone (GnRH) stimulation test, and comprehensive judgment of imaging examination [1, 2]. A case-control study was conducted between the two groups according to the principle of strict age matching. According to the inclusion/exclusion criteria, 58 girls with CPP and 58 girls with PT were finally recruited as the case group. For the control group, 124 healthy, age-matched girls were recruited from the general public in Henan. Questionnaire data on the risk factors for PP and enzyme-linked immunoassay (ELISA) and electrochemical luminescence method results based on peripheral blood were used for the final statistical analysis. This study was approved by the ethics committee of the Children's Hospital affiliated to Zhengzhou University (approval number: 2021-K-053), and informed consent was obtained from the patients' guardians.

### 2.4. Questionnaire

The questionnaire was designed to survey the potentially influential factors associated with PP. Questions were related to patients' name, sex, date of birth, date of diagnosis, place of residence, parents' career and educational background, mother's menarche age, time spent daily in front of an electronic screen, outdoor activity time, dietary status, consumption of dietary supplements, off-season fruit consumption, high-calorie food consumption, processed meat consumption, daily sleep time, daily sleep duration, use of night lights, frequency of use of plastic products, use of adult cosmetics, history of early development in siblings hazardous substance exposure history, time spent with parents, and history of secondhand smoke exposure. The relevant parameters were considered to occur “frequently” if they occurred seven or more times per week.

### 2.5. Physical Examination and Auxiliary Examination

Height, body weight (BW), abdominal circumference, hip circumference, breast and pubic hair development, Tanner staging, and cardiopulmonary/liver/spleen examination were included in the physical examination. The auxiliary examination included liver and kidney function testing, fundamental values of sex hormones and a provocation test, 25-hydroxyvitamin D levels, thyroid function test, and insulin-like growth factor-1 measurement using fasting serum samples. Other examinations included BA, gonadal color Doppler ultrasound, and pituitary magnetic resonance imaging (only for girls with CPP).

### 2.6. Laboratory Analyses

Fasting peripheral blood samples were drawn in the morning, and the serum was isolated. The MT level (D711355, Sangon Biotech, Shanghai), serotonin level (D751013, Sangon Biotech, Shanghai), leptin level (D711368, Sangon Biotech, Shanghai), and kisspeptin level content (ml060071, Ml Bio, Shanghai) in serum were measured by ELISA according to the manufacturer's instructions. LH level, FSH level, E2 level, and vitamin D level were measured by chemiluminescence according to the Immunology Department of Henan Children's Hospital.

### 2.7. Statistical Analysis

GraphPad Prism 8.01 software (GraphPad Software, San Diego, CA, USA) was used for all analyses. The characteristics of the study population were described using frequency distributions for categorical variables and mean and standard deviation values, medians, and ranges for continuous variables, depending on whether the data were normally distributed or not. Continuity variables are normally distributed by skewness analysis. The statistical significance of the continuous variable comparisons was assessed using one-way analysis of variance (ANOVA), and Dunnett's multiple comparisons test, a statistical comparison procedure to compare each treatment group with a single control group, was used for multiple comparisons. Data with unequal variance were compared using Tamhane's T2 test. Logistic multiple regression analysis was used for the analysis of PP-related factors. All statistical tests were two-tailed, and differences with a *p* value of <0.05 were considered statistically significant.

## 3. Results and Discussion

### 3.1. Changes in the Number of Female New-Onset PP Outpatients

**Figure 1:**
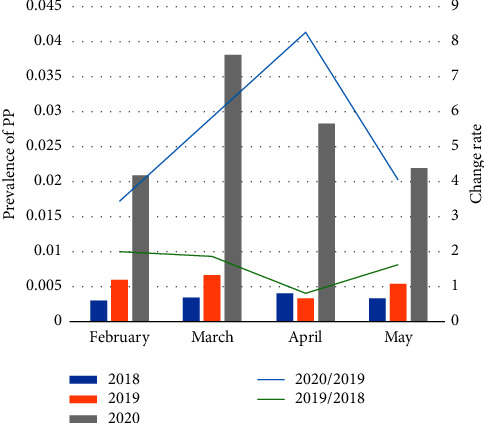
Prevalence and change rate in the number of female cases of new-onset precocious puberty in 22 medical institutions in different time periods with a CR of <0.5 to identify inhibited diseases and CR of >3 to identify stimulated diseases (CR = ratio of the prevalence of female cases of new-onset precocious puberty in different time periods).

Data from 22 medical institutions showed that 4281 female patients were diagnosed with new-onset PP between February and May 2020. This number was 5.01 times that observed in the same period in 2018 (855 patients) and 3.14 times that observed in the same period in 2019 (1346 patients). These differences were statistically significant (*p* < 0.01). The prevalence of new-onset PP increased from 0.40% in 2018 to 6.23% in 2020 (Figure 1). The CR for new-onset PP between 2020 and 2019 from February to May was 3.44–8.26, demonstrating that the COVID-19 pandemic in 2020 was a risk factor for the general increase in the incidence of PP in different regions of Henan Province. The CR for new-onset PP was 0.87–1.99 between 2019 and 2018 from February to May.

**Figure 2:**
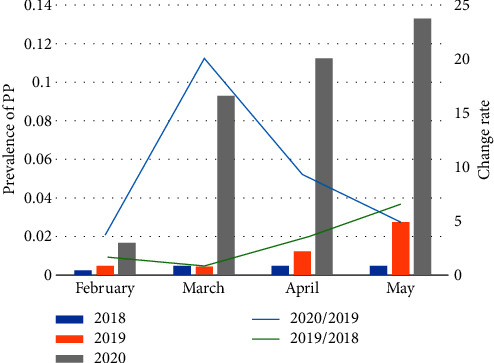
Prevalence and change rate in the number of female cases of new-onset precocious puberty in Henan Children's Hospital in different time periods with a CR of <0.5 to identify inhibited diseases and CR of >3 to identify stimulated diseases (CR = ratio of the prevalence of female cases of new-onset precocious puberty in different time periods).

The number of cases of female new-onset PP observed in Henan Children's Hospital/Children's Hospital affiliated to Zhengzhou University from February to May 2020 (2141 visits) was 20.01 times than that observed in the same period in 2018 (107 visits) and 6.21 times more than that in the same period in 2019 (345 visits). The prevalence increased from 0.41% in 2018 to 9.88% in 2020 (Figure 2). The CR between 2020 and 2019 from February to May was 3.75–19.97 in Henan Children's Hospital, which was consistent with the results of this multicenter study. The CR between 2019 and 2018 from February to May was 0.88–6.43, while the CR was 1 between April 2019 and April 2018 and 2 between May 2019 and May 2018 (Figures 1 and 2).

### 3.2. Analysis of the Risk Factors for PP

The general characteristics of the three groups are summarized in Table 1, including chronological age (CA), BA, height, BW, body mass index (BMI), and the rates of obesity and overweightness.

The CPP and PT groups presented with a significantly older BA than did the control group (*p* < 0.0001 and *p*=0.0163, respectively), although the three groups had the same mean CA. The height, BW, and BMI values in the CPP and PT groups were significantly higher than those in the control group (control vs. CPP and control vs. PT, both *p* < 0.0001).

**Figure 3:**
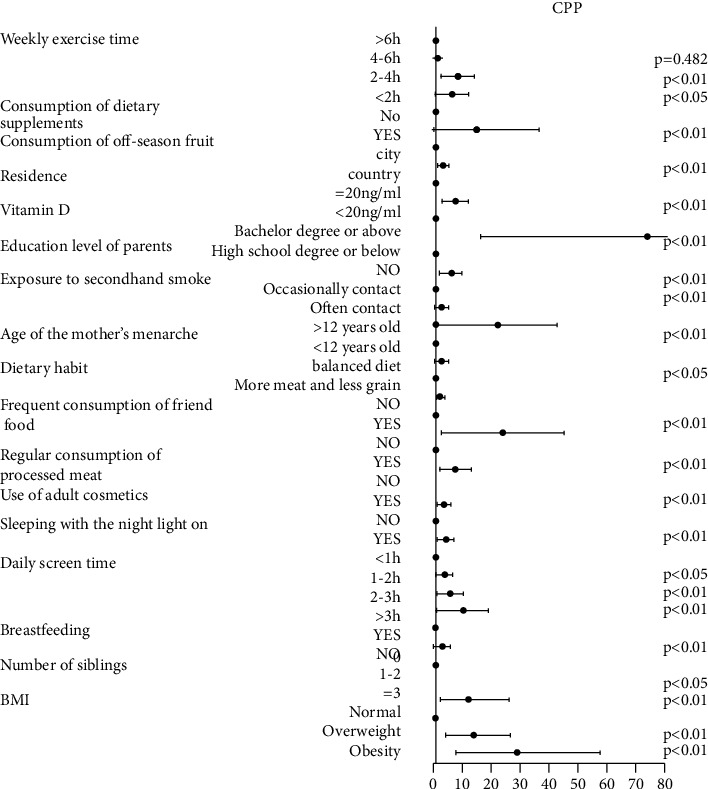
Logistic regression analysis of the risk factors for CPP. Multivariate odds ratios and 95% confidence intervals from unconditional logistic regression models are used in the analysis. CPP, central precocious puberty.

**Figure 4:**
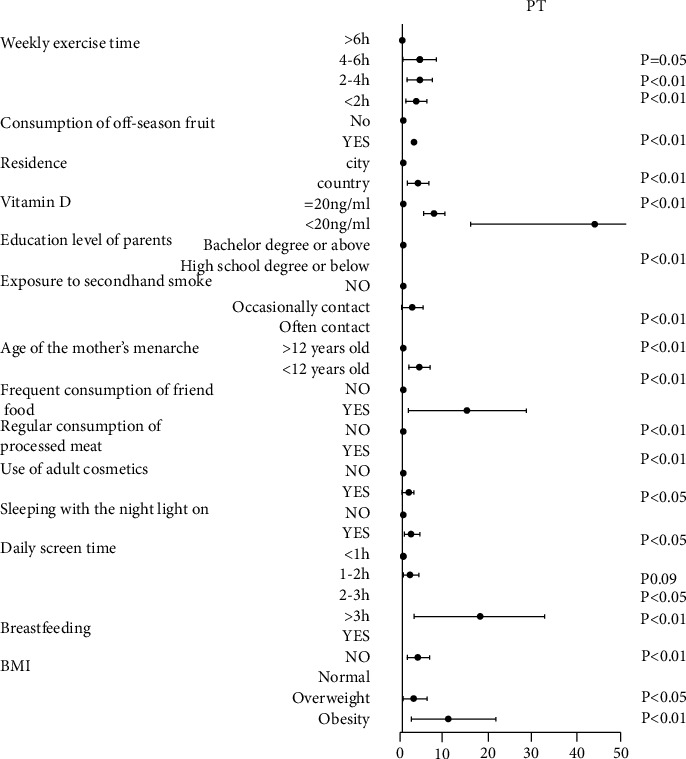
Logistic regression analysis of the risk factors for PT. Multivariate odds ratios and 95% confidence intervals from unconditional logistic regression models are used in the analysis. PT, premature thelarche.

Based on the data from the questionnaire, multifactor logistic regression analysis showed that many parameters were high-risk factors for PP (Figures 3 and 4), including use of electronic devices for prolonged periods, less exercise time, higher BMI, vitamin D deficiency, younger age of mother's menarche, frequent use of a night light, frequent use of adult cosmetics, consumption of fried food and processed meat, exposure to secondhand smoke, residence in a rural setting, consumption of off-season fruits, consumption of stimulants, and lower education level of parents. Among these, the following were highly independent risk factors for PP, as determined by their multivariate odds ratios (ORs; given in parentheses): vitamin D deficiency (≤20 ng/ml; CPP: 59.283, PT: 38.058), obesity (CPP: 21.382, PT: 8.253), consumption of processed meat (CPP: 6.225, PT: 6.225), exposure to secondhand smoke (CPP: 15.619, PT: 17.571), and use of electronic devices for prolonged periods (>3 h/d; CPP: 7.5, PT: 14.5).

### 3.3. Related Hormone Measurements (Table 2)

There were significant differences in the sex hormone, serotonin, MT, leptin, and kisspeptin levels among the three groups (*p* < 0.05). Luteinizing hormone (LH), follicle stimulation hormone (FSH), estrogen (E2), and leptin levels were significantly higher in the CPP group than in the control and PT groups (*p* < 0.05). FSH, E2, and leptin levels were significantly higher in the PT group than in the control group (*p* < 0.05). However, there was no significant difference between the PT and control groups in terms of LH levels (*p*=0.365). Serotonin levels were significantly higher in the control group than in the CPP and PT groups (*p* < 0.05), but there was no significant difference in serotonin levels between the PT and CPP groups (*p*=0.412). Kisspeptin levels were significantly higher in the CPP and PT groups than in the control group, but there was no significant difference in kisspeptin levels between the CPP and PT groups (*p*=0.946). The MT levels were significantly higher in the PT group than in the control group; however, these levels were not significantly different between the CPP and PT groups or between the CPP and control groups (both *p* > 0.05).

## 4. Discussion

The 22 medical units included in this study are located in geographically dispersed regions of Henan Province; hence, this study provides a comprehensive and accurate reflection of the prevalence of PP in Henan Province. The results of this study showed that the incidence of new-onset female PP increased significantly between 2018 and 2020 and between 2019 and 2020. The minimum CR value was >3, demonstrating that home quarantine due to the COVID-19 pandemic was a high-risk factor for the occurrence of PP in girls. Our findings are consistent with those of other studies [9, 10]. Therefore, we can conclude that the COVID-19 pandemic has had a significant impact on children's lifestyles, causing an increase in the incidence of PP among girls.

Several studies have shown that PP is related to obesity and being overweight [8, 9, 14]. The mechanism [15, 16] for these factors leading to early puberty may be related to the influence of adipokines (particularly leptin and adiponectin) on the HPG axis, the expression of adipokines and their receptors in the gonads, and the peripheral activities of adipose tissue (such as through other adipose factors and aromatase activity). The obesity gene in white fat stimulates the synthesis and release of leptin, and high levels of leptin stimulate the secretion of kisspeptin [17, 18]. Kisspeptin is a peptide product of the *KISS1* gene. It is considered a key gatekeeper for the activation of GnRH neurons and the reproductive axis in adolescence. It is a powerful stimulator of GnRH-induced gonadotropin secretion [19]. Serum kisspeptin levels were positively correlated with BMI [20]. During the pandemic, long-term home quarantine, less time for outdoor exercise, and frequent fried food consumption during the Chinese Spring Festival caused rapid growth in children, along with an increased BMI. This study showed that the incidences of obesity and overweightness in the CPP and PT groups were significantly higher than those in the control group. BMI increased significantly in both the CPP and PP groups compared with that in the control group; the BMI in the CPP group was higher than that in the PT group, which further confirmed that obesity or overweightness could lead to PP. Furthermore, the leptin and kisspeptin levels were significantly higher in the CPP group than in the control and PT groups, which is consistent with the finding of a higher BMI in the PP group than in the control and PT groups. Therefore, during the pandemic, the increase in PP in girls may be related to the increase in leptin and kisspeptin levels caused by changes in diet during home isolation, reduced outdoor activities, and increased BMI. Studies have reported that the activation of mutations or polymorphisms in the *KISS1* gene may be associated with PP [13, 21], indicating that PP has a genetic tendency. This study also showed that the age of mothers at menarche was related to PP incidence in girls. Therefore, genetic testing is recommended for children with familial PP.

This study showed that the long-term use of electronic devices was closely related to the occurrence of PP. Due to the impact of the pandemic and long-term home quarantine, outdoor activities were reduced and time spent on online learning and video game-times increased. Children in the CPP and CP groups spent a significantly longer time using electronic devices than those in the control group. Prolonged exposure to artificial light sources (blue light), including smartphones, tablets, and laptops, causes inhibition of the secretion of MT in teenagers [22, 23]. MT is a hormone that regulates the sleep-wake cycle. It exhibits rhythmic changes during the day and night, and its secretion is inhibited by light [24–26].

MT receptors are expressed in the hypothalamus, pituitary gland, and ovaries [27–29] and have a regulatory effect on the hypothalamus-pituitary-ovarian axis. MT binds to hypothalamic receptors and inhibits the secretion of GnRH, thereby inhibiting the initiation of the gonadal axis [30]. Animal studies have confirmed that oral MT can inhibit the occurrence of PP [31]. Furthermore, serotonin, the precursor of MT, was significantly lower in the CPP group than in the control group. We speculated that the increase in time spent on electronic devices inhibited the secretion of serotonin, which led to the reduction in MT levels and promoted the occurrence of PP. MT is a protective agent in the human body. It is used to treat sleep disorders [32], is an antioxidant, and has anti-inflammatory and immunomodulatory effects on many diseases [33–36]. MT has been used for patients with severe coronary pneumonia during the COVID-19 pandemic [37]. Although multiple short-term and long-term randomized, double-blind, controlled trials have confirmed that an oral dose of MT within the range of 2–10 mg/day is safe for children and adolescents [38], further studies are required to determine whether MT can be used for the prevention of PP. Although this was contrary to our expectations, the small absolute value of MT may have little biological significance. This lower measured value may also be related to the sensitivity of the detection method. It is possible that the 6-hydroxysulfate MT present in morning urine is more representative of MT secretion in the body than that of the MT levels in urine collected at other times. Therefore, further investigation is required to determine whether the prolonged use of electronic devices can cause a decrease in the secretion of MT and is therefore a potential mechanism of PP.

This study suggested that vitamin D deficiency is an independent risk factor for PP. Vitamin D deficiency may affect the absorption of calcium ions, and calcium regulates vitamin D receptors, further activating the HPG axis, leading to PP [39, 40]. Furthermore, in the process of tryptophan synthesis of serotonin, tryptophan hydroxylase-2 can be activated by vitamin D; therefore, vitamin D deficiency can lead to a decrease in serotonin synthesis, which further affects MT synthesis [41].

The questionnaire used in this study was designed to investigate a variety of factors, such as genetics, nutrition, lifestyle, and diet, in an attempt to explain the obvious increase in the prevalence of PP. The questionnaire showed that the use of adult skin care products was related to PP and may be related to exposure to exogenous estrogen.

The incidence of PP was higher in rural areas than in urban areas. This finding is consistent with Liu's epidemiological survey results, and the reason may be related to the existence of environmental endocrine disruptors [12]. The presence of high-density farms and factories in the suburbs leads to a greater possibility of children living in these areas being exposed to environmental endocrine disruptors. PP may also be related to an unhealthy diet, including frequent fast-food consumption in rural areas. The results of our study showed that living in a rural area was an independent risk factor for PP.

This study had some limitations. In terms of risk factor assessment, the number of participants was relatively small; moreover, there was no way to obtain validated questionnaire-based data for 2018 and 2019, which may have better reflected the reasons for the increased incidence of PP. In addition, the present study only demonstrated the prevalence of PP; further studies evaluating the incidence in a larger sample are necessary.

## 5. Conclusions

In summary, the increased incidence of PP in girls during the COVID-19 pandemic may be related to prolonged use of electronic devices, changes in diet, decreased outdoor activities, increased BMI, genetic factors, and endocrine disruptors. Vitamin D deficiency, inhibition of serotonin and MT secretion, and increased secretion of leptin and kisspeptin are involved in the occurrence of PP. Therefore, increasing outdoor activities, a balanced diet, vitamin D supplementation, and reducing the use of electronic devices are all conducive to the prevention of PP.

## Figures and Tables

**Table 1:** The characteristics of patients in the precocious puberty groups and the control group.

Variable	Control	CPP	PT	*P*
Population number	124	58	58	—
CA (years)	7.21 ± 0.91	7.31 ± 1.00	7.27 ± 0.96	NS
BA (years)	7.13 ± 0.93	8.91 ± 1.32	7.63 ± 1.48	0.001
Height (cm)	123.32 ± 7.87	132.76 ± 7.01	129.46 ± 7.87	0.001
BW (kg)	22.77 ± 4.77	32.93 ± 6.57	28.66 ± 5.90	0.001
BMI (kg/m^2^)	15.07 ± 2.20	18.02 ± 2.36	17.04 ± 2.78	0.001
Obesity (percentage)	7.26	22.41	25.86	0.001
Overweight (percentage)	4.03	22.41	12.07	0.001

CPP, central precocious puberty; PT, premature thelarche; CA, chronological age; BA, bone age; BW, body weight; BMI, body mass index; NS, not significant.

**Table 2:** Hormone levels in the study population.

Variable	Control	CPP	PT	*P*
Population number	124	58	58	
Serotonin (ng/mg)	11.55 ± 4.33	9.99 ± 5.48	8.73 ± 4.05	<0.001
Melatonin (pg/mg)	0.17 ± 0.06	0.19 ± 0.09	0.21 ± 0.10	0.002
Leptin (ng/mg)	0.21 ± 0.07	0.36 ± 0.07	0.28 ± 0.08	<0.001
Kisspeptin (pg/mg)	309.55 ± 85.30	354.17 ± 129.34	352.87 ± 105.66	0.005
LH (mIU/mL)	0.11 ± 0.04	1.96 ± 2.50	0.15 ± 0.09	<0.001
FSH (mIU/mL)	1.77 ± 0.74	3.48 ± 1.91	2.49 ± 1.45	<0.001
E2 (pg/mL)	11.72 ± 9.44	30.12 ± 18.71	19.00 ± 15.38	<0.001
Vitamin D (ng/mL)	27.61 ± 8.64	13.76 ± 4.10	15.26 ± 6.11	<0.001

CPP, central precocious puberty; PT, premature thelarche; LH, luteinizing hormone; FSH, follicle stimulation hormone; E2, estrogen.

## Data Availability

The data that support the findings of this study are available from Dr. Dongxia Fu (fudongxiaaaa@163.com).

## References

[1] Kletter G. B., Klein K. O., Wong Y. Y. (2015). A pediatrician’s guide to central precocious puberty. *Clinical Pediatrics*.

[2] Subspecialty Group of Endocrinologic (2015). Hereditary and metabolic diseases, the society of pediatrics, Chinese medical association; editorial board, Chinese journal of pediatrics. “Consensus statement for the diagnosis and treatment of central precocious puberty (2015)”. *Zhonghua Er Ke Za Zhi*.

[3] Buck Louis G. M., Gray L. E., Marcus M. (2008). Environmental factors and puberty timing: expert panel research needs. *Pediatrics*.

[4] Głąb E., Wikiera B., Bieniasz J., Barg E. (2016). The influence of GnRH analog therapy on growth in central precocious puberty. *Advances in Clinical and Experimental Medicine*.

[5] Willemsen R. H., Elleri D., Williams R. M., Ong K. K., Dunger D. B. (2014). Pros and cons of GnRHa treatment for early puberty in girls. *Nature Reviews Endocrinology*.

[6] Kim Y. J., Kwon A., Jung M. K. (2019). Incidence and prevalence of central precocious puberty in korea: an epidemiologic study based on a national database. *The Journal of Pediatrics*.

[7] Bräuner E. V., Busch A. S., Eckert-Lind C., Koch T., Hickey M., Juul A. (2020). Trends in the incidence of central precocious puberty and normal variant puberty among children in Denmark, 1998 to 2017. *JAMA Network Open*.

[8] Wei H., Chen Y., Li C. (2010). Epidemiological investigation of precocious puberty in children aged 3 to 12 in Zhengzhou. *Journal of Applied Clinical Pediatrics*.

[9] Liu Y., Yu T., Li X. (2021). Prevalence of precocious puberty among Chinese children: a school population-based study. *Endocrine*.

[10] Li H., Yu G., Duan H., Fu J., Shu Q. (2020). Changes in children’s healthcare visits during coronavirus disease-2019 pandemic in hangzhou, China. *The Journal of Pediatrics*.

[11] Stagi S., De Masi S., Bencini E. (2020). Increased incidence of precocious and accelerated puberty in females during and after the Italian lockdown for the coronavirus 2019 (COVID-19) pandemic. *Italian Journal of Pediatrics*.

[12] Chen Y., Wang Y., Ding G. (2018). Association between bisphenol a exposure and idiopathic central precocious puberty (ICPP) among school-aged girls in Shanghai, China. *Environment International*.

[13] Li D., Wu Y., Cheng J. (2020). Association of polymorphisms in the kisspeptin/GPR54 pathway genes with risk of early puberty in Chinese girls. *The Journal of Cinical Endocrinology and Metabolism*.

[14] Lian Q., Mao Y., Luo S. (2019). Puberty timing associated with obesity and central obesity in Chinese Han girls. *BMC Pediatrics*.

[15] Reinehr T., Roth C. L. (2019). Is there a causal relationship between obesity and puberty?. *The Lancet Child & Adolescent Health*.

[16] Nieuwenhuis D., Pujol-Gualdo N., Arnoldussen I. A. C., Kiliaan A. J. (2020). Adipokines: a gear shift in puberty. *Obesity Reviews*.

[17] Michalakis K., Mintziori G., Kaprara A., Tarlatzis B. C., Goulis D. G. (2013). The complex interaction between obesity, metabolic syndrome and reproductive axis: a narrative review. *Metabolism*.

[18] Machinal-Quelin F., Dieudonne M. N., Pecquery R., Leneveu M. C., Giudicelli Y. (2002). Direct in vitro effects of androgens and estrogens on ob gene expression and leptin secretion in human adipose tissue. *Endocrine*.

[19] Navarro V. M., Castellano J. M., García-Galiano D., Tena-Sempere M. (2007). Neuroendocrine factors in the initiation of puberty: the emergent role of kisspeptin. *Reviews in Endocrine & Metabolic Disorders*.

[20] Kaya C., Alay İ, Babayeva G. (2019). Serum Kisspeptin levels in unexplained infertility, polycystic ovary syndrome, and male factor infertility. *Gynecological Endocrinology*.

[21] Silveira L. G., Noel S. D., Silveira-Neto A. P. (2010). Mutations of the KISS1 gene in disorders of puberty. *Journal of Clinical Endocrinology and Metabolism*.

[22] Park H. R., Choi S. J., Jo H., Cho J. W., Joo E. Y. (2020). Effects of evening exposure to light from organic light-emitting diodes on melatonin and sleep. *Journal of Clinical Neurology*.

[23] Mortazavi S. A. R., Parhoodeh S., Hosseini M. A. (2018). Blocking short-wavelength component of the visible light emitted by smartphones’ screens improves human sleep quality. *J Biomed Phys Eng*.

[24] Dibner C., Schibler U., Albrecht U. (2010). The mammalian circadian timing system: organization and coordination of central and peripheral clocks. *Annual Review of Physiology*.

[25] Reiter R. J., Rosales-Corral S., Coto-Montes A. (2011). The photoperiod, circadian regulation and chronodisruption: the requisite interplay between the suprachiasmatic nuclei and the pineal and gut melatonin. *Journal of Physiology & Pharmacology*.

[26] Welsh D. K., Takahashi J. S., Kay S. A. (2010). Suprachiasmatic nucleus: cell autonomy and network properties. *Annual Review of Physiology*.

[27] Odo M., Koh K., Takada T. (2014). Changes in circadian rhythm for mRNA expression of melatonin 1A and 1B re-ceptors in the hypothalamus under a neuropathic pain-like state. *Synapse*.

[28] Bae S. E., Wright I. K., Wyse C. (2014). Regulation of pituitary MT1 melatonin receptor expression by gonadotrophin-releasing hormone (GnRH) and early growth response factor-1 (Egr-1): in vivo and in vitro studies. *PLoS One*.

[29] Jablonska K., Pula B., Zemla A. (2014). Expression of the MT1 melatonin receptor in ovarian cancer cells. *International Journal of Molecular Sciences*.

[30] Sizonenko P. C., Lang U., Aubert M. L. (1982). Neuroendocrinology of puberty. Role of melatonin in man. *Annales d’Endocrinologie*.

[31] Jeon G. H., Kim H. J., Park J., Lee S. H., Cheon Y. P., Choi D. (2020). The effects of daily melatonin gavage on reproductive activity in the male Syrian hamsters. *Dev Reprod*.

[32] Izuhara M., Kawano K., Otsuki K., Hashioka S., Inagaki M. (2021). Prompt improvement of difficulty with sleep initiation and waking up in the morning and daytime somnolence by combination therapy of suvorexant and ramelteon in delayed sleep-wake phase disorder: a case series of three patients. *Sleep Medicine*.

[33] Manchester L. C., Coto-Montes A., Boga J. A. (2015). Melatonin: an ancient molecule that makes oxygen metabolically tolerable. *Journal of Pineal Research*.

[34] Galano A., Tan D. X., Reiter R. J. (2011). Melatonin as a natural ally against oxidative stress: a physicochemical examination. *Journal of Pineal Research*.

[35] Esalatmanesh K., Loghman A., Esalatmanesh R. (2021). Effects of melatonin supplementation on disease activity, oxidative stress, inflammatory, and metabolic parameters in patients with rheumatoid arthritis: a randomized double-blind placebo-controlled trial. *Clinical Rheumatology*.

[36] Ashrafizadeh M., Najafi M., Kavyiani N., Mohammadinejad R., Farkhondeh T., Samarghandian S. (2021). Anti-inflammatory activity of melatonin: a focus on the role of NLRP3 inflammasome. *Inflammation*.

[37] Ameri A., Asadi M. F., Kamali M. (2021). Evaluation of the effect of melatonin in patients with COVID-19-induced pneumonia admitted to the Intensive Care Unit: a structured summary of a study protocol for a randomized controlled trial. *Trials*.

[38] Parvataneni T., Srinivas S., Shah K., Patel R. S. (2020). Perspective on melatonin use for sleep problems in autism and attention-deficit hyperactivity disorder: a systematic review of randomized clinical trials. *Cureus*.

[39] Pérez-Fernandez R., Alonso M., Segura C., Munoz I., Garcia-Caballero T., Diguez C. (1997). Vitamin D receptor gene expression in human pituitary gland. *Life Sciences*.

[40] Nicholas C., Davis J., Fisher T. (2016). Maternal vitamin D deficiency programs reproductive dysfunction in female mice offspring through adverse effects on the neuroendocrine axis. *Endocrinology*.

[41] Patrick R. P., Ames B. N. (2015). Vitamin D and the omega 3 fatty acids control serotonin synthesis and action, part 2: relevance for ADHD, bipolar disorder, schizophrenia, and impulsive behavior. *The FASEB Journal*.

